# RETRACTED: Yadav et al. A Feature Extraction Using Probabilistic Neural Network and BTFSC-Net Model with Deep Learning for Brain Tumor Classification. *J. Imaging* 2023, *9*, 10

**DOI:** 10.3390/jimaging12110536

**Published:** 2026-10-28

**Authors:** Arun Singh Yadav, Surendra Kumar, Girija Rani Karetla, Juan Carlos Cotrina-Aliaga, José Luis Arias-Gonzáles, Vinod Kumar, Satyajee Srivastava, Reena Gupta, Sufyan Ibrahim, Rahul Paul, Nithesh Naik, Babita Singla, Nisha S. Tatkar

**Affiliations:** 1 Department of Computer Science, University of Lucknow, Lucknow 226007, Uttar Pradesh, India; 2 Department of Computer Application, Marwadi University, Rajkot 360003, Gujrat, India; 3 School of Computer, Data and Mathematical Sciences, Western Sydney University, Penrith, NSW 2751, Australia; 4 Department of Investigation, Universidad Privada San Juan Bautista, Chorrillos 15067, Peru; 5 Department of Business, Pontificia Universidad Católica del Perú, Av. Universitaria 1801, San Miguel 15088, Peru; 6 Department of Computer Applications, ABES Engineering College, Ghaziabad 201009, Uttar Pradesh, India; 7 Department of Computer Science and Engineering, University of Engineering and Technology Roorkee, Roorkee 247667, Uttarakhand, India; 8 Department of Pharmacognosy, Institute of Pharmaceutical Research, GLA University, Mathura 281406, Uttar Pradesh, India; 9 Neuro-Informatics Laboratory, Department of Neurological Surgery, Mayo Clinic, Rochester, MN 55905, USA; 10 Department of Radiation Oncology, Massachusetts General Hospital, Harvard Medical School, Boston, MA 02115, USA; 11 iTRUE (International Training and Research in Uro-Oncology and Endourology) Group, Manipal 576104, Karnataka, India; 12 Department of Mechanical and Industrial Engineering, Manipal Institute of Technology, Manipal Academy of Higher Education, Manipal 576104, Karnataka, India; 13 Curiouz TechLab Private Limited, BIRAC-BioNEST, Manipal Government of Karnataka Bioincubator, Manipal 576104, Karnataka, India; 14 Chitkara Business School, Chitkara University, Chandigarh 140401, Punjab, India; 15 Department of Postgraduate Diploma in Management, Institute of PGDM, Mumbai Education Trust, Mumbai 400050, Maharashtra, India

The journal retracts the article titled “A Feature Extraction Using Probabilistic Neural Network and BTFSC-Net Model with Deep Learning for Brain Tumor Classification” [1], cited above.

Following publication, concerns were brought to the attention of the Editorial Office regarding the accuracy of the authorship list and origins of this study [1].

In accordance with the journal’s standard procedures, the Editorial Office and Editorial Board conducted an investigation. Although the authors cooperated during the investigation, the information provided for evaluation was still deemed insufficient to verify the accuracy of the authorship list, authors’ contributions or confirm the provenance of the study. As a consequence, the Editorial Board has lost confidence in the integrity of this publication and decided to retract the article in accordance with MDPI’s retraction policy.

This retraction was approved by the Editor-in-Chief of the *Journal of Imaging*.

The authors have been informed of this retraction and have indicated their disagreement with this decision.
